# Targeted Therapies in Sarcomas: Challenging the Challenge

**DOI:** 10.1155/2012/626094

**Published:** 2012-06-03

**Authors:** Juan Martín Liberal, Laura Lagares-Tena, Miguel Sáinz-Jaspeado, Silvia Mateo-Lozano, Xavier García del Muro, Oscar M. Tirado

**Affiliations:** ^1^Laboratori d'Oncología Molecular, Institut d'Investigació Biomèdica de Bellvitge (IDIBELL), L'Hospitalet de Llobregat, 08908 Barcelona, Spain; ^2^Nanomedicine Research Program, Molecular Biology and Biochemistry Research Center, CIBBIM-Nanomedicine, Vall d'Hebron Hospital Research Institute, 08035 Barcelona, Spain

## Abstract

Sarcomas are a heterogeneous group of mesenchymal malignancies that very often lead to death. Nowadays, chemotherapy is the only available treatment for most sarcomas but there are few active drugs and clinical results still remain very poor. Thus, there is an imperious need to find new therapeutic alternatives in order to improve sarcoma patient's outcome. During the last years, there have been described a number of new molecular pathways that have allowed us to know more about cancer biology and tumorigenesis. Sarcomas are one of the tumors in which more advances have been made. Identification of specific chromosomal translocations, some important pathways characterization such as mTOR pathway or the insulin-like growth factor pathway, the stunning development in angiogenesis knowledge, and brand new agents like viruses have lead to the development of new therapeutic options with promising results. This paper makes an exhaustive review of preclinical and clinical evidence of the most recent targeted therapies in sarcomas and provides a future view of treatments that may lead to improve prognosis of patients affected with this disease.

## 1. Introduction

 Sarcomas are a rare and heterogeneous group of malignant tumors of mesenchymal origin. They can occur through all the lifespan and affect patients of all ages, although certain specific subtypes are more frequent in children and young adults. Almost every part of the body may be involved, bone and soft-tissue being the most typical place of arising. Sarcomas are associated with bad prognosis and approximately 50% of all patients develop metastases even if they are diagnosed at early stages. Lungs are the most frequent site of dissemination and metastases are the final cause of death in almost all these patients [1]. These high mortality levels make sarcomas one of the most challenging tumors in oncology.

For most sarcomas, chemotherapy is currently the only available treatment. Unfortunately, a very limited number of useful drugs are active against this disease and responses used to be poor and short. In fact, advanced-stage patients treated with the most active drugs in this disease (anthracyclines and ifosfamide) achieve only a median survival of around 1 year [2]. Thus, it is necessary to identify new agents to improve therapy for patients affected by this often mortal condition.

In the last years, great advances have been made in the understanding of sarcomas' molecular biology [3]. Consequently, new targeted compounds have been developed and tested in order to improve efficacy and outcome achieved with classic drugs. This paper will extensively review the most relevant pathways in soft tissue and bone sarcomas and the preclinical and clinical experience with the most recent targeted therapies.

## 2. Angiogenesis

 In the last years, angiogenesis has been one of the most studied processes in tumor biology with interesting results. Patients with several malignancies such as renal cancer or colorectal cancer are currently being treated with angiogenic inhibitors alone or in combination with conventional chemotherapy. These patients achieve significant improvement in overall survival (OS) and/or progression-free survival (PFS) [4]. Sarcomas have been recently added to the group of tumors in which angiogenesis is known to play an important role.

 One of the key effectors in angiogenesis is the vascular endothelial growth factor (VEGF). It is well known that the interaction between VEGF and its receptor 2 (VEGF-R2) is the most crucial step in angiogenesis [5–7] and there are some studies that relate VEGF with sarcomas. A study by Potti et al. published in 2004 correlated VEGF serum levels with outcome in patients with sarcoma [8]. 68 out of 273 patients (24.9%) included in the study showed VEGF overexpression. The most common sarcomas in which VEGF overexpression was detected were malignant fibrous histiocytoma (30%), carcinosarcoma (30%), leiomyosarcoma (25%), and dermatofibrosarcoma (20%) but VEGF overexpression had prognostic value only in patients affected with leiomyosarcoma. In addition, this was associated with a shorter survival. Graeven et al. also determined VEGF serum levels in 85 patients with STS before surgery. They found a very strong correlation between tumor grade and VEGF serum levels, the poorest differentiated tumors being the ones with the highest VEGF levels [9]. Another study was designed to assess the correlation between VEGF and tumor grade [10]. Results confirmed that tumor grade correlated with VEGF expression. Furthermore, 78% of patients who died of sarcoma progression had high VEGF levels. However, VEGF expression was not found to be an independent predictor of OS or disease-free survival (DFS). In contrast, a study by Iyoda et al. that correlated VEGF overexpression and survival in patients with soft-tissue sarcomas (STSs) of the thorax found this correlation statistically significant. Thus, patients with absent or faint VEGF expression had higher 5-year DFS than patients with a strong VEGF expression (83.3% versus 13.2%, resp.) [11].

 Hypoxia-inducible factor1*α* (HIF-1*α*) is another important player in angiogenesis since it is a transcription factor that acts as an upstream regulator of VEGF. In a 2006 paper by Shintani et al. HIF-1*α* expression was determined by immunohistochemistry in a group of 49 specimens of STS. The analysis showed that patients with a strong or moderate expression of HIF-1*α* had poorer OS than those with a weak or negative expression [12].

 There are many other angiogenesis markers but not all of them are so clearly related with prognosis and survival as VEGF and HIF-1*α* in sarcomas. Microvascular density (MVD) is one of them. Thus, in a work by Comandone et al., patients with high MVD had worse OS and DFS than patients with lower MVD [13]. In contrast, 3 other authors reported results that did not confirm this correlation between MVD and prognosis, so the real role of MVD in sarcomas remains unclear [14–16].

### 2.1. Angiogenic Inhibitors

**Figure 1:**
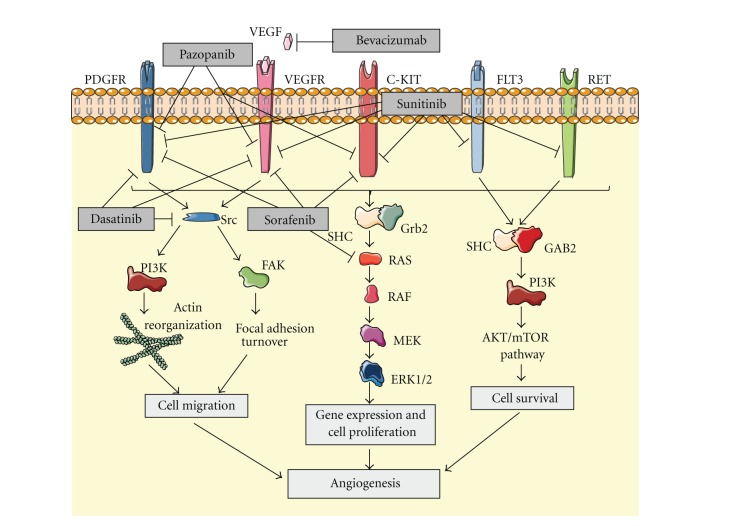
Inhibition of angiogenesis by blocking different tumorigenic signaling pathways.

These previous data provided a rationale for the development of preclinical and clinical studies with angiogenic inhibitors in sarcomas. An increasing number of these drugs have been developed in the last years and the effects of many of them have been assessed (Figure 1).

#### 2.1.1. Small-Molecule VEGF-R Inhibitors

Sunitinib is a multitargeted tyrosine kinase inhibitor (TKI) active against VEGF-R1, 2, 3, PDGFR and KIT, among others. In 2009, Stacchiotti et al. reported 3 responses and 1 stabilization in a cohort of 5 patients affected with advanced alveolar STS treated with sunitinib [17]. The same year, a phase II trial of sunitinib in the treatment of non-GIST sarcomas was published. A cohort of 53 patients with advanced non-GIST STS received 37.5 mg of sunitinib daily. 10 of these patients (20%) achieved stable disease (SD) for at least 16 weeks and, interestingly, 1 patient affected with desmoplastic round cell tumor (DSRCT) achieved a durable partial response (PR) for 56 weeks [18]. Focusing on 3 specific histologies (leiomyosarcoma, liposarcoma, and malignant fibrous histiocytoma), a phase II study was reported in 2010. In this trial, 48 patients with unresectable or metastatic STS of the histologies mentioned previously were treated daily with 50 mg of sunitinib malate for 4 weeks every 6 weeks. 3 or less prior lines of therapy were allowed. Median PFS and OS for liposarcoma, leiomyosarcoma, and fibrous histiocytoma were 3.9 and 18.6, 4.2 and 10.1, and 2.5 and 13.6 months, respectively. The 3-month progression-free rates (PFRs) in the untreated and pretreated patients with liposarcoma, leiomyosarcoma, and fibrous histiocytoma were 75% and 69.2%, 60%, and 62.5%, and 25% and 44.4%, respectively. The authors concluded that the 3-month PFR of >40% suggests activity for sunitinib at least in liposarcomas and leiomyosarcomas [19]. In contrast to these studies, the Gynecologic Oncology Group conducted a phase II study to assess the efficacy of sunitinib in the treatment of recurrent or persistent uterine leiomyosarcoma with disappointing results: of 25 patients enrolled, just 2 achieved a PR and the median PFS was 1.5 months [20].

 Sorafenib is another TKI recently added to the group of drugs with activity in sarcomas. This TKI targets VEGF-R 2 and 3, PDGFR, Raf, and KIT, and several preclinical studies have demonstrated efficacy in different soft-tissue and bone sarcoma cell lines [20–25]. These encouraging preclinical results, together with some case reports of responses in patients with sarcomas treated with sorafenib [26, 27], led to the development of several clinical trials. In 2009, Maki et al. published a phase II trial of sorafenib in patients with metastatic or recurrent sarcomas [28]. A total of 145 patients with different types of sarcomas were enrolled but just the angiosarcoma patients met the response rate (RR) primary end point planned for the study (5 out of 37 patients, 14%). Another study pointed out osteosarcoma as other sarcoma subtype in which sorafenib showed activity. The Italian group conducted a phase II trial of sorafenib in relapsed and unresectable high-grade osteosarcoma after failure of standard therapy that showed 3 PRs (8%), 2 minor responses (6%), and 12 SDs (34%). Furthermore, median PFS and OS were 4 and 7 months, respectively, demonstrating sorafenib as the first targeted therapy active in osteosarcoma [29]. In order to find other responsive histologies to sorafenib, the Southwest Oncology Group (SWOG) promoted a phase II trial testing its activity in advanced vascular sarcoma, high-grade liposarcoma, and leiomyosarcoma [30]. No responses were found and just 6 out of 8 patients with vascular sarcoma achieved clinical benefit. New strategies, such as discontinuation treatment, have also been tested. Thus, Pacey et al. assessed the efficacy of sorafenib in STS in a phase II randomized discontinuation trial [31]. Evidence of activity was found, since some tumor regressions were observed including 1 objective partial response.

Pazopanib is a new TKI that also has effects on angiogenesis by blocking VEGF-R 1, 2, and 3, PDGFR, and KIT. A phase II trial performed by the European Organisation for Research and Treatment of Cancer (EORTC) in patients with relapsed or refractory advanced STS treated with pazopanib achieved promising results [32]. 142 patients were enrolled and they were classified in 4 different groups: adipocytic STS, leiomyosarcomas, synovial sarcomas, and other STS types. Pazopanib in adipocytic STS patients showed insufficient activity but PFR at 12 weeks in the remaining groups was encouraging: 18 (44%) of 41 patients in the leiomyosarcoma cohort, 18 (49%) of 37 in the synovial sarcomas, and 16 (39%) of 41 in the other STS types. These data led to the first placebo-controlled randomized phase III trial to date with a VEGFR inhibitor (pazopanib) in advanced STS. The results, reported in the 2011 ASCO Annual Meeting, achieved a significant increase in PFS showing the relevance of angiogenesis in sarcomas [33]. Finally, the activity of a new TKI named dasatinib has been assessed in several sarcoma cell lines. This drug blocks VEGF-R2 and PDGFR and has the unique property of inhibiting the Src family. This latter effect leads to the inhibition of migration and invasion in different sarcoma cell lines *in vitro*, which can lead to further development of clinical trials [34–37].

#### 2.1.2. Anti-VEGF Antibodies

Bevacizumab is the only monoclonal anti-VEGF antibody to date that has been proved to have activity in sarcomas. A set of preclinical studies demonstrated activity in sarcoma models *in vitro* and *in vivo* [38–42]. The experience achieved in a number of other malignancies such as colon, breast, or non-small-cell lung cancer, has shown that the effect of bevacizumab in combination with chemotherapy is higher than as a single agent. This strategy was first assessed in sarcomas by D'Adamo el al. in a phase II trial of doxorubicin and bevacizumab in patients with metastatic STS [43]. There were just 2 partial responses but 11 of 17 patients recruited (65%) achieved stable disease for 4 cycles or more. Interestingly, cardiac toxicity was significantly high and 4 patients developed cardiac toxicity grade 2, 1 patient grade 3, and 1 patient grade 4 despite prophylactic treatment with dexrazoxane. These results suggest the activity of this combination in sarcomas but make necessary finding safer schedules. The efficacy of bevacizumab in combination with other drugs has also been explored in a recently published phase IB trial. In this study, 38 chemotherapy-naive patients with advanced or recurrent STS were treated with a combination of docetaxel, gemcitabine, and bevacizumab [44]. After a median follow-up of 36 months, the overall RR observed was 31.4%. There were 5 complete responses (CRs), 6 PRs, and 18 SDs lasting for a median of 6 months. The combination treatment was considered safe and the highest-grade adverse events found were mostly related to bevacizumab.

## 3. The Insulin-Like Growth Factor Pathway

**Figure 2:**
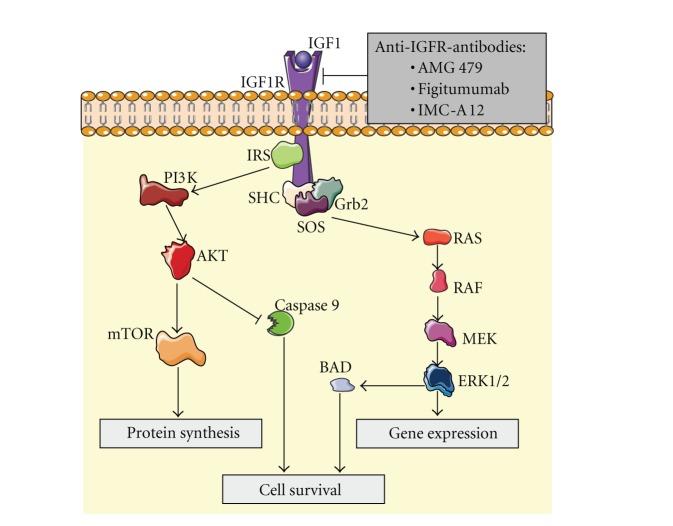
The activation of IGFR by ligand binding triggers a signal transduction pathway that causes the activation of mTOR pathway and inhibition of apoptosis, thus promoting cell survival. Anti-IGFR antibodies prevent this effect.

 The insulin-like growth factor (IGF) system is a well-known complex network that regulates growth and development in superior organisms (Figure 2). The IGF receptor type 1 (IGFR-1) belongs to the family of tyrosine kinase receptors. The binding of its ligand IGF1 causes its phosphorylation and the subsequent activation of the downstream pathway that finally leads to proliferation and inhibition of apoptosis. The relationship between the IGF system and sarcomas is long-time known but the first studies to describe it were merely epidemiological [45]. In the last years, a variety of studies have confirmed this point. Thus, Prieur et al. described in 2004 the binding of EWS/FLI1 to the IGFBP3 promoter and the subsequent inhibition of IGFBP3 and the increase in free IGFR-1 ligand levels, which are related to the development of this malignancy [46]. Other sarcomas such as alveolar soft part sarcoma, leiomyosarcoma, synovial sarcoma, rhabdomyosarcoma, or desmoplastic small round cell tumor have also been described as tumors in which increases in IGFR-1 levels or some of its ligands have been correlated with sarcomagenesis [47–58].

### 3.1. IGF Pathway Inhibitors

The background described previously has led to the development of several studies that test different strategies to assess the inhibition of the IGF pathway in sarcoma models *in vitro* and* in vivo *[59–67]. The results, especially in rhabdomyosarcoma and Ewing's sarcoma, have been encouraging and the clinical development of these drugs is currently being carried out.

#### 3.1.1. Anti-IGFR-1 Antibodies

Among the different strategies developed to inhibit the IGF pathway, the most promising results have been achieved with monoclonal antibodies. There are several phase I studies that assess the safety and efficacy of inhibiting the IGF system in sarcomas with these drugs. Thus, Tolcher et al. published in 2009 an early clinical study with AMG 479, a fully human monoclonal antibody to IGFR-1. 15 out of 53 patients enrolled were sarcoma patients (12 Ewing's sarcoma, 3 others). Interestingly, 1 durable CR and 1 PR were achieved in 2 patients with Ewing's sarcoma [68]. In another phase I study recently published, a cohort of patients with different sarcoma histologies were treated with figitumumab (a fully human monoclonal antibody targeting the IGFR-1). Among 29 patients enrolled, 2 Ewing's sarcoma patients had objective responses (1 CR, 1 PR), 6 Ewing's sarcoma patients, 1 synovial sarcoma, and 1 fibrosarcoma achieved SD [69]. Unfortunately, the clinical development of this drug has been stopped due to disappointing results in other malignancies.

Several phase II trials with anti-IGFR-1 antibodies are currently being conducted. Preliminary data of treatment with IMC-A12 (cixutumumab) in patients with advanced or metastatic STS and Ewing's sarcoma have been reported in the 2011 ASCO Annual Meeting. The best results were observed in the adipocytic sarcoma arm, with clinical benefit being achieved in 22 out of 37 patients (1 PR, 21 SD). Moreover, the PFS at 12 weeks in this group of patients was 50% [70]. With such promising results, further investigations are warranted.

#### 3.1.2. IGFR-1 TKIs

A number of small molecules that inhibit IGFR-1 by binding to the tyrosine kinase intracellular portion of the receptor are currently in clinical development. No results of efficacy and safety are available yet but there is a body of preclinical data that support this therapeutic approach [71–78]. Reports of clinical outcome in patients treated with these drugs are long awaited and will allow us to confirm the usefulness of this strategy.

## 4. The Mammalian Target Rapamycin Pathway

**Figure 3:**
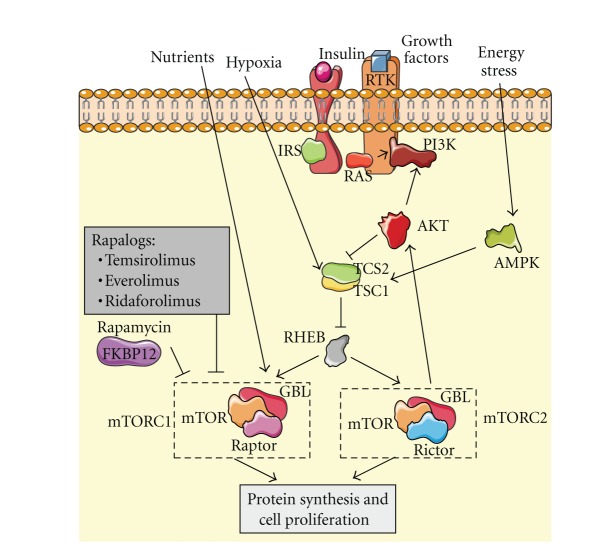
AKT activation, among other stimulus, can activate mTOR complexes, mTORC1 and mTORC2, promoting protein synthesis and cell proliferation. Rapamycin and rapalogs are specific inhibitors of mTORC1 but an escape route remains through mTORC2 activity.

 The mammalian target of rapamycin (mTOR) is a serine/threonine kinase integrated in the phosphatidyl-inositol 3-kinase (PI3K) complex network of signaling. It forms part of 2 multiprotein complexes named mTOR complex 1 and mTOR complex 2 (mTORC1 and mTORC2) and plays a key role in cell growth, proliferation, angiogenesis, and survival (Figure 3). Due to the many functions that mTOR regulates, its abnormal activity leads to a number of malignancies including sarcomas. The upregulation of growth factors or mutations in tyrosine kinase receptors that belongs to the mTOR network have been reported to be involved in the development of various sarcomas [79–84]. Furthermore, deletions of some mTOR pathway tumor suppressors such as tuberous sclerosis complex 1 and 2 (TSC1 and TSC2) and neurofibromatosis type 1 (NF1) are associated with both, benign and malignant mesenchymal tumors [85–88]. Hence, the capital importance of mTOR in tumorigenesis has made the development of mTOR inhibitors an important issue in oncology.

### 4.1. mTOR Inhibitors

To date, 4 compounds with anti-mTOR activity have reached the clinical setting. All of them belong to a single family of drugs and are derived from an initial molecule called sirolimus (rapamycin). Thus, temsirolimus, everolimus, ridaforolimus, and the already mentioned sirolimus are nowadays under investigation in sarcomas and other tumors. Sirolimus inhibits mTOR kinase activity by binding to FK506 binding protein (FKBP12), one of the proteins that form mTORC1. This leads to cell cycle arresting in G1 phase and the subsequent inhibition in proliferation and cell growth. Preclinical data suggest activity of sirolimus in some paediatric malignancies including Ewing's sarcoma, rhabdomyosarcoma, and osteosarcoma [89, 90]. But, despite preclinical evidences, the only phase II trial with sirolimus in sarcomas to date (combined with ciclofosfamide) has been reported as negative [91]. A prodrug of sirolimus named temsirolimus has also been tested in treatment of sarcoma. At least 2 papers have reported tumor growth inhibition in murine xenograft models of rhabdomyosarcoma when treated with temsirolimus [92, 93]. But the first phase II trial published reported disappointing results, with just 2 out of 41 STS patients achieving PR. Thus, the authors concluded that temsirolimus in patients with STS has limited clinical activity and significant toxicity [94]. On the other hand, preliminary results of another phase II trial of temsirolimus in pediatric patients with neuroblastoma, high-grade glioma, and rhabdomyosarcoma are slightly more encouraging, with 2 PR (1 neuroblastoma, 1 rhabdomyosarcoma) and 11 SD from a total of 52 patients [95]. Everolimus is an orally available mTOR inhibitor developed to be much more soluble than sirolimus. Cell cycle arrest in different tumor models has been observed with everolimus and even prolonged survival in a murine model of leiomyosarcoma has been reported [96]. The only preliminary data reported to date of a phase II trial of everolimus in STS or bone sarcoma showed a clinical efficacy (CR + PR + SD) of 20% [97]. The last member of the family of rapamycin analogs (also called rapalogs) is ridaforolimus. It has a better pharmacokinetic profile, presenting a more favorable bioavailability than sirolimus. Activity of ridaforolimus alone has been confirmed in cell lines and xenograft models of sarcoma. Moreover, additive inhibitory effects when combined with cytotoxic agents have also been reported [98]. In the clinical setting, a phase II trial by Chawla et al. achieved a 29% clinical benefit in patients with advanced sarcoma treated with intravenous ridaforolimus and a median OS of 40 weeks [99]. Such interesting results led to the only phase III trial to date with an mTOR inhibitor: the SUCCEED trial. This double-blind, placebo-controlled phase III trial randomized sarcoma patients who had achieved CR, PR, or SD after 1, 2, or 3 lines of chemotherapy to receive placebo or ridaforolimus as maintenance treatment. Results recently reported showed a 28% reduction in the risk of progression in ridaforolimus arm compared with placebo arm and a 3.1-week improvement in PFS [100]. Mature OS data are not yet available but everything indicates that ridaforolimus is a promising drug in treatment of sarcomas and further investigations are warranted.

A very rare type of mesenchymal malignant tumor named Perivascular Epithelioid Cell Tumor (PEComa) has been specially related to the mTOR pathway. Dysfunction in tumor suppressors TSC1 and 2 and the subsequent upregulation of mTORC1 seems to be a crucial step in the development of this disease. Thus, responses with sirolimus and temosirolimus have been reported in at least 2 studies [101, 102]. However, another study did not find these positive results, making necessary additional investigations [103].

All 4 rapalogs described previously belong to a first generation of mTOR inhibitors able to inhibit mTORC1 but not mTORC2. mTORC2 seems to be responsible for feedback phosphorylation of Akt in the PI3K/Akt pathway, which could be a possible mechanism of resistance in sarcomas. So, a new generation of mTOR inhibitors with activity against mTORC1 and mTORC2 is under early development in an attempt to block this escape route.

## 5. Specific Chromosomal Translocations as Therapeutic Targets

 About 1/3 of sarcomas are associated with specific chromosomal translocations. These translocations are an early step in carcinogenesis, promoting some of the processes that finally lead to the appearance of sarcomas [104]. Thus, trying to inhibit the effects of these genetic alterations seems to be a reasonable option in fighting sarcomas. Dermatofibrosarcoma protuberans is an example of this group of sarcomas. It is characterized by a t(17; 22) translocation that leads to the overexpression of platelet-derived growth factor B (PDGFB). Imatinib is a TKI with known activity against the receptor of PDGFB, PDGFR. Because of that, its efficacy has been assessed in dermatofibrosarcoma protuberans with excellent results (46% PR, 25% SD) [105, 106].

Clear cell sarcoma is also associated with a specific chromosomal translocation, t(12; 22)(q13; q12) in most of the cases. One of the consequences of this chromosomal rearrangement is the activation of the hepatocyte growth factor receptor (MET). This activation is involved in invasion and angiogenesis. A response in a clear cell sarcoma patient with the MET inhibitor ARQ197 has recently been reported in a phase II trial, which is especially relevant in this treatment-resistant disease [107].

But probably the most studied translocation-related sarcoma is Ewing's sarcoma. This disease characteristically has a t(11; 22) translocation that leads to expression of the oncogenic fusion protein EWS/FLI1. This chimerical protein is involved in Ewing's sarcoma development since it acts as an oncogenic transcription factor but needs binding to other proteins such as RNA helicase A for its oncogenic function. Recently described YK-4-279, a new compound that blocks RNA helicase A binding to EWS/FLI1, induces apoptosis in Ewing's sarcoma cell lines and reduces tumor growth in orthotopic xenografts [108]. A clinical trial to assess the efficacy of this new drug is planned.

## 6. Virotherapy

One of the most innovative approaches in cancer treatment developed in the last years is oncolytic virotherapy. The general basis of this strategy is that the therapeutic virus is capable to recognize specifically tumor cells, replicate into them, and lead to their death without damaging normal cells. A paper recently published in Nature by Breitbach et al. assessed the safety and efficacy of an intravenous delivery of an oncolytic poxvirus in humans. Treatment was generally well tolerated and the only sarcoma patient enrolled (a 55-year-old female with a heavily pretreated advanced leiomyosarcoma) achieved SD by RECIST criteria for >16 weeks [109]. This result is as encouraging for sarcoma treatment as a 2010 study published by Li et al. In that paper, the authors reported marked cytolysis and apoptosis in osteosarcoma cell lines *in vitro* and significant tumor growth suppression in a human osteosarcoma murine xenograft model when treated with a telomerase-specific oncolytic adenovirus [110]. More in-deep preclinical and clinical investigations are needed but virotherapy seems a feasible and reasonable option in future treatments for sarcomas.

## 7. Combined Targeted Therapies

 Although clinical experience with targeted therapies in sarcomas as single treatment is short, there are some published studies with combination of 2 of these drugs. The most studied double-inhibition-targeted therapies are those related with IGFR-1 pathway, with preclinical evidences of activity in sarcoma models [111–113]. Based on these studies, at least 2 phase I trials have been reported, one of them with figitumumab and everolimus in advanced sarcoma patients and the other solid tumors and the other with cixutumumab combined with temsirolimus in Ewing's sarcoma patients that is currently enrolling [114, 115]. Both studies reported good toxicity profiles, making the combination of 2 targeted therapies an attractive and safe option to be developed. Most trials involving the targeted therapies reviewed in this paper are summarized in Table 1.

## 8. Future Perspectives

 An increasing number of new targets in treatment of sarcomas are being identified in the last years. The finding of new important key effectors in sarcomas biology has resulted in a growing development of inhibitor drugs that need to be tested. For instance, increased activity in Hedgehog pathway has recently been reported in certain sarcomas such as rhabdomyosarcoma, osteosarcoma, chondrosarcoma, and Ewing's sarcoma [117–121]. Other reports suggest that rhabdomyosarcoma and osteosarcoma aggressiveness seems to be related with Notch signalling pathway [122, 123]. This has led to the conduction of a clinical trial with Hedgehog and Notch inhibitors with no results to date.

 Anaplastic lymphoma kinase (ALK) is another protein recently related to sarcomas that is upregulated in approximately 50% of cases of inflammatory myofibroblastic tumor. Interestingly, in a phase I trial with the ALK inhibitor crizotinib, a patient with ALK overexpression related to inflammatory myofibroblastic tumor experienced a durable PR [116].

Histone deacetylase (HDAC) inhibitors have also shown signs of efficacy in preclinical models of sarcomas [124–128] and several clinical trials are currently ongoing.

PI3K/mTOR dual-inhibitor NVP-BEZ235 that induces G1 cell cycle arrest in sarcomas *in vitro* and *in vivo* [129] has been identified as well as a brand new promising agent.

 Other molecules such as MDM2 and protein families BCL-2 and CDKs have been described lately as associated with sarcomas [130–132]. Future investigations with inhibitors are warranted.

## 9. Conclusions

 Despite the low incidence of sarcomas in general population regarding other types of cancer, the finding of new active treatments is essential since it is a rarely curable disease. Hence, a rapidly increasing number of targeted therapies have been developed in the last years with different results. In general, most of these treatments do not achieve significant tumor shrinkage and SD is usually the best response reported. In addition, OS has not been dramatically increased in the majority of these patients, showing the necessity of keep working. In an attempt to improve response rates, one of the strategies that are currently ongoing is the combination treatment with targeted therapies and conventional cytotoxic drugs. Toxicity is an important issue when using this approach and more clinical trials are needed to assess the safety of this therapeutic option. Combinations of more than one targeted agent are another reasonable choice. The strategy of inhibiting a signalling pathway and simultaneously others that could be possible ways of escape is an attractive alternative still under early development. Phase I trials reported to date with 2 targeted therapies show favourable toxicity profiles, making this strategy a feasible and promising issue to be explored. Different approaches like those, future identification of new pathways and their correspondent inhibitors and the arise of innovative agents such as oncolytic viruses, make the final endpoint of cure sarcsomas a goal not so far to be reached.

## Figures and Tables

**Table 1:** Targets, drugs, and clinical trials. Drugs and their specific targets in sarcoma treatment and clinical trials with their results: positive (+), negative (−), or not published (x).

	Target	Drug	Clinical trial	
Vascular endothelial growth factor	Tyrosine kinase inhibitors	Sunitinib	+	Phase II trial: non:GIST sarcoma [18].
			+	Phase II trial: leiomyosarcoma, liposarcoma, and malignant fibrous histiocytoma. [19].
			−	Phase II study: treatment of recurrent/persistent uterine leiomyosarcoma [20].
		Sorafenib	+	Phase II trial: patients with metastatic or recurrent sarcomas [28].
			+	Phase II trial: efficacy of sorafenib in STS [31].
			+	Phase II trial: relapsed and unresectable high-grade osteosarcoma [29].
			+	Phase II trial: advanced vascular sarcoma, high grade liposarcoma and leiomyosarcoma [30].
		Pazopanib	+	Phase II trial: patients with relapsed or refractory advanced STS [32].
			+	Phase III trial: placebo-controlled randomized trial in advanced STS [33].
		Dasatinib	x	No results published.
	Anti-VEGF antibodies	Bevacizumab	+	Phase II trial: doxorubicin and bevacizumab in patients with metastatic STS [43].
			+	Phase IB trial: efficacy of bevacizumab in combination with other drugs [44].

Insulin-like growth factor	Tyrosine kinase inhibitors	Clinical development	x	No results published.
	Anti-IGFR1 antibodies	AMG 479	+	Phase I trial: response of sarcoma patients to a fully monoclonal antibody to IGFR-1 [68].
		Figitumumab	−	Phase I trial: different sarcoma patients [70].
		IMC-A12 (cixutumumab)	+	Phase II trial: patients with advanced or metastatic STS and Ewing Sarcoma [91].

Mammalian target of rapamycin	mTORC1 tyrosine kinase inhibitors	Sirolimus	−	Phase II trial: combination with cyclophosphamide in sarcomas [91].
		Temsirolimus	+	Phase II trial: temsirolimus in pediatric patients with neuroblastoma, high-grade glioma, and rhabdomyosarcoma [95].
			−	Phase II trial: treatment in STS patients [94]
			+	Phase II trial: everolimus in STS or bone sarcoma [97]
		Everolimus	+	Phase II trial: treatment in patients with advanced bone and soft tissue sarcomas [99].
		Ridaforolimus	+	Phase III trial: placebo-controlled randomized sarcoma patient [99].

Hepatocyte growth factor receptor	MET inhibitors	ARQ197	+	Phase II trial: patients with microph thalmia transcription-family-(MiT-) associated tumors [107].
Virus	Tumor cell	Oncolytic poxvirus	+	Phase I trial: oncolytic poxvirus in different cancer patients [109].
Insulin-like growth factor/ mammalian target of rapamycin	Tyrosine kinase inhibitors	Figitumumab/ everolimus	+	Phase I trial: advanced sarcoma patients and other solid tumors [114].
		Cixutumumab/ temsirolimus	+	Phase I trial: patients with advanced cancer including sarcomas [115].

Other targets	ALK inhibitors	Crizotinib	+	Phase I trial: inflammatory myofibroblastic tumor with ALK overexpression [116].
	HDAC inhibitors		+	Ongoing clinical trials.
	PI3K/mTOR	NVP-BEZ235	+	Ongoing clinical trials.
